# Interpretation of multiple probe sets mapping to the same gene in Affymetrix GeneChips

**DOI:** 10.1186/1471-2105-8-13

**Published:** 2007-01-15

**Authors:** Maria A Stalteri, Andrew P Harrison

**Affiliations:** 1 Departments of Biological Sciences and Mathematical Sciences, University of Essex, Wivenhoe Park, Colchester, Essex CO4 3SQ, UK

## Abstract

**Background:**

Affymetrix GeneChip technology enables the parallel observations of tens of thousands of genes. It is important that the probe set annotations are reliable so that biological inferences can be made about genes which undergo differential expression. Probe sets representing the same gene might be expected to show similar fold changes/z-scores, however this is in fact not the case.

**Results:**

We have made a case study of the mouse Surf4, chosen because it is a gene that was reported to be represented by the same eight probe sets on the MOE430A array by both Affymetrix and Bioconductor in early 2004. Only five of the probe sets actually detect Surf4 transcripts. Two of the probe sets detect splice variants of Surf2. We have also studied the expression changes of the eight probe sets in a public-domain microarray experiment. The transcripts for Surf4 are correlated in time, and similarly the transcripts for Surf2 are also correlated in time. However, the transcripts for Surf4 and Surf2 are not correlated. This proof of principle shows that observations of expression can be used to confirm, or otherwise, annotation discrepancies.

We have also investigated groups of probe sets on the RAE230A array that are assigned to the same LocusID, but which show large variances in differential expression in any one of three different experiments on rat. The probe set groups with high variances are found to represent cases of alternative splicing, use of alternative poly(A) signals, or incorrect annotations.

**Conclusion:**

Our results indicate that some probe sets should not be considered as unique measures of transcription, because the individual probes map to more than one transcript dependent upon the biological condition. Our results highlight the need for care when assessing whether groups of probe sets all measure the same transcript.

## Background

One of the most widely used microarray platforms is the Affymetrix GeneChip. A GeneChip consists of a quartz wafer to which are attached some 500,000 different 25-mer deoxyoligonucleotides, which are known as probes [1]. Gene expression is measured by extracting mRNA from the cells or tissues of interest and hybridising the mRNA sample to the 25-mer probes on the microarray. Each expressed transcript is represented on an array by a series of probe pairs known as a probe set [1]. Each pair consists of a perfect match probe, with its 25-base sequence identical to the gene of interest, and a mismatch probe, whose sequence is the same as the perfect match except for position thirteen, where the base is set to the complementary of the perfect match. The mismatch probe was introduced by Affymetrix as a measure of cross-hybridisation. Each probe set on the Affymetrix MOE430A and RAE230A arrays consists of 11 probe pairs, and is given a unique identifier consisting of a seven digit number, followed by the optional characters _s, _a, or _x, and ending in _at [2,3].

During the array design process [2,3] Affymetrix collects sequences and annotations from various public databases including GenBank, dbEST and RefSeq. The sequences are aligned to the draft genome assembly for the relevant organism in order to assess sequence orientation and quality. The orientation of a sequence is determined by using consensus splice sites from genome alignments, detected polyadenylation sites, and coding sequence and EST read direction annotations. UniGene [4] is used to create initial seed clusters of cDNA sequences, which are further subdivided into subclusters representing distinct transcripts. A consensus sequence is a nucleotide sequence assembled by Affymetrix based on all the member sequences in a subcluster [5]. Probes are usually selected from the 600-base region at the 3' end of the consensus sequence. When there are alternative polyadenylation sites less than 600 bases apart only the probe selection region on the upstream polyadenylation site is used. The target sequence for a probe set is the part of the consensus sequence that the probes are taken from, starting with the first base of the most 5' probe and ending with the twenty-fifth base of the most 3' probe.

The aim of most gene expression microarray experiments is to obtain a list of genes which are differentially expressed under certain conditions. For the cases of multiple probe sets representing the same gene, the assumption would be that the expression levels should be upregulated or downregulated together. However, although this is the fundamental assumption behind microarray technology, we observe that this is not always the case. In order to elucidate the causes of these discrepancies we examined several cases where the expression levels of probe sets representing the same gene are not correlated, and we believe we have determined at least some of the likely reasons for this behaviour for the cases we examined.

## Results

### The annotation of Surf4

**Figure 1:**
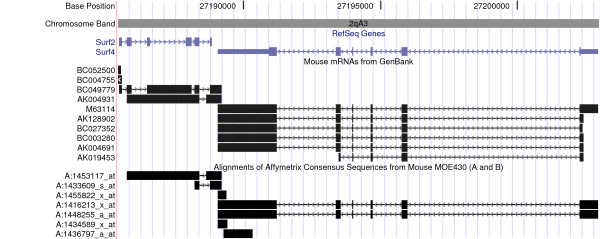
Screen shot of the UCSC Genome Browser on October 2003 mouse assembly, showing Surf2 and Surf4 on chromosome 2 [10]. The black or blue boxes represent exons. The arrows in the intronic regions indicate the direction of transcription.

The mouse surfeit 4 gene, gene symbol Surf4, maps to the same eight probe sets in the Affymetrix NetAffx records for the MOE430A array (April 2004 release) [6,7] and in the Bioconductor [8] annotations for moe430a (March 2004 release) [9]. Table 1 lists the 8 probe sets and the chromosomal alignments of the Affymetrix target and consensus sequences for each probe set. Table 2 details how the annotation of the probe sets by Bioconductor has changed with time. We find a similar time-dependence in some of the Affymetrix annotations (data not shown) [6,7,11]. The mouse Surf4 gene is on the reverse strand of chromosome 2 (Figure 1). However, only five of the probe sets were correctly assigned to the reverse strand of chromosome 2, whereas two probe sets align to the forward strand of chromosome 2 and one probe set aligns to chromosome 19. The 2 probe sets which align to the forward strand of chromosome 2 are incorrectly assigned and should be assigned to Surf2, LocusID 20931.

**Table 1:** Chromosomal alignment of target and consensus sequences for Surf4 probe sets on the MOE430A array.

**Probe Set ID**	**Chromosomal Alignment of Affymetrix Target Sequence [7]**	**Chromosomal Alignment of Affymetrix Consensus Sequence [10]**
1453117_at	chr2:27,188,365 – 27,188,758(+)	chr2: 27,185,730 – 27,189,203(+)
1433609_s_at	chr2:27,188,260 – 27,189,050(+)	chr2: 27,188,216 – 27,189,206(+)
1455822_x_at	chr2:27,189,082 – 27,189,245(-)	chr2: 27,189,060 – 27,189,354(-)
1416213_x_at	chr2:27,189,911 – 27,190,390(-)	chr2: 27,189,060 – 27,202,949(-)
1448255_a_at	chr2:27,189,344 – 27,189,640(-)	chr2: 27,189,061 – 27,202,949(-)
1434589_x_at	chr2:27,189,129 – 27,189,301(-)	chr2: 27,189,062 – 27,189,367(-)
1436797_a_at	chr2:27,189,904 – 27,190,264(-)	chr2: 27,189,247 – 27,190,320(-)
1427285_s_at	chr19:5,657,821 – 5,658,039(-)	chr19: 5,657,707 – 5,660,083(-)

Chromosomal coordinates are from the UCSC October 2003 mouse assembly [10].

**Table 2:** Gene symbol, LocusID/GeneID, and UniGene cluster ID for the 8 Surf4 probe sets from six successive releases of the Bioconductor moe430a annotation package [9].

**Probe set ID**	**Mar/04**	**Sept/04**	**Jan/05**	**May/05**	**Oct/05**	**Apr/06**
1416213_x_at	Surf4	Surf4	Surf4	Surf4	Surf4	Surf4
	20932	20932	20932	20932	20932	20932
	Mm.196863	Mm.300594	Mm.300594	Mm.300594	Mm.300594	Mm.300594
1436797_a_at	Surf4	Surf4	Surf4	Surf4	Surf4	Surf4
	20932	20932	20932	20932	20932	20932
	Mm.196863	Mm.300594	Mm.300594	Mm.300594	Mm.300594	Mm.300594
1448255_a_at	Surf4	Surf4	Surf4	Surf4	Surf4	Surf4
	20932	20932	20932	20932	20932	20932
	Mm.196863	Mm.300594	Mm.300594	Mm.300594	Mm.300594	Mm.300594
1434589_x_at	Surf4	Surf4	Surf4	Surf4	Surf4	Surf4
	20932	20932	20932	20932	20932	20932
	Mm.196863	Mm.300594	Mm.300594	Mm.300594	Mm.300594	Mm.300594
1455822_x_at	Surf4	Surf4	Surf4	Surf4	Surf4	Surf4
	20932	20932	20932	20932	20932	20932
	Mm.196863	Mm.300594	Mm.300594	Mm.300594	Mm.300594	Mm.300594
1433609_s_at	Surf4	Surf4	Surf4	Surf4	Surf4	Surf4
	20932	20932	20932	20932	20932	20932
	Mm.196863	Mm.300594	Mm.300594	Mm.300594	Mm.300594	Mm.300594
1453117_at	Surf4	Surf4	Surf4	NA	Surf2	Surf2
	20932	20932	20932	NA	20931	20931
	Mm.196863	Mm.300594	Mm.300594	NA	Mm.6874/Mm.300594	Mm.6874/Mm.300594
1427285_s_at	Surf4	Surf4	Surf4	Ramp2	Malat1	Malat1
	20932	20932	20932	54409	72289	72289
	Mm.196863	Mm.300594	Mm.300594	Mm.298256/Mm.358667	Mm.298256	Mm.298256

**Figure 2:**
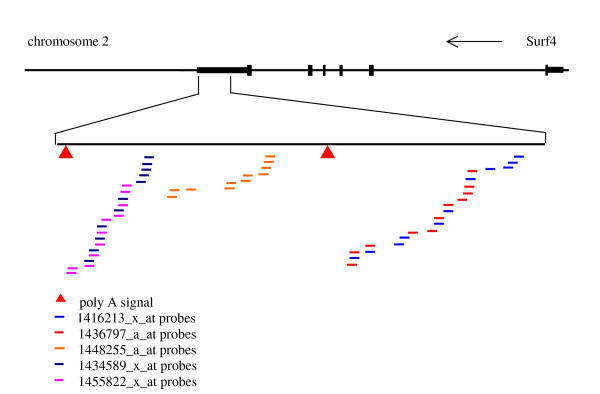
Mapping of individual probes in probe sets 1416213_x_at (blue lines), 1436797_a_at (red lines), 1448255_a_at (orange lines), 1434589_x_at (dark blue lines) and 1455822_x_at (pink lines) to the 3' UTR of Surf4 exon 6. The arrow shows the direction of transcription. Probe sequences were obtained from Affymetrix [7]. Genomic sequence of mouse chromosome 2 was from the UCSC May 2004 assembly [10]. The alignment of Surf4 transcript sequences to the genomic sequence was obtained from UCSC Genome Bioinformatics [10]. Chromosomal coordinates of individual probes were obtained from Ensembl version 28 [15].

Surf4 and Surf2 are part of the surfeit gene locus, an unusual cluster of six housekeeping genes on chromosome 2, which are not related either by sequence or function [12]. Adjacent genes in the cluster are transcribed in opposite directions. Figure 1 shows a screen shot of the UCSC Genome Browser [10,13,14] on the region of mouse chromosome 2 containing Surf2 and Surf4. Surf4 specifies two major mRNAs, a more abundant transcript of 2.8 kb and a less abundant transcript of 2.0 kb [12]. The poly(A) addition signals are in the 3' UTR, at 2468 – 2474 for the 2.0 kb transcript and at 3257 – 3262 for the 2.8 kb transcript. The 3' end of the 2.8 kb transcript overlaps the 3' end of Surf2 by 133 base pairs. The 5 Surf4 probe sets were mapped to the genomic sequence of chromosome 2 as shown in Figure 2. All 5 probe sets mapped to the 3' UTR region on exon 6 of Surf4. Note that probe sets 1416213_x_at and 1436797_a_at have 4 probes in common, and probe sets 1434589_x_at and 1455822_x_at have 6 probes in common. Probe sets 1416213_x_at and 1436797_a_at would detect both the 2.0 kb and 2.8 kb Surf4 transcripts, whereas probe sets 1448255_a_at, 1434589_x_at and 1455822_x_at would detect only the longer 2.8 kb transcript.

**Figure 3:**
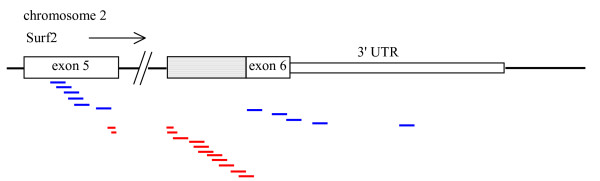
Mapping of individual probes in probe sets 1433609_s_at (blue lines) and 1453117_at (red lines) to mouse genomic sequence. The arrow shows the direction of transcription. The diagram shows exon 5 and exon 6 of Surf2, with the 3' UTR as described by Williams and Fried [17]. The shaded box represents the alternative splice acceptor site for exon 6. Probe sequences were obtained from Affymetrix [7]. Genomic sequence of mouse chromosome 2 was from the UCSC October 2003 assembly [10]. The alignment of Surf2 transcript sequences to the genomic sequence was obtained from UCSC Genome Bioinformatics [10].

Table 1 shows that the target and consensus sequences for probe sets 1433609_s_at and 1453117_at align to the + strand of chromosome 2. However, the screen shot of the UCSC Genome Browser in Figure 1 indicates that Surf4 is transcribed from the – strand, so the assignment of these 2 probe sets to Surf4 is incorrect. This error can probably be traced to the GenBank record for the mRNA AK004931 [16], the transcript which Affymetrix gives as the Representative Public Identifier for probe set 1453117_at [7]. The revision history for AK004931 shows that the term 'surfeit gene 4' was added to the description of the sequence given in the definition line (defline) on 26-Dec-2001, but was changed to 'product: surfeit gene 2' on 05-Dec-2002. Figure 1 shows that the AK004931 sequence aligns to Surf2. Surf2 gives rise to two alternatively spliced transcripts, which differ in the splice acceptor site used for the start of exon 6 [17]. As shown in Figure 3, probe set 1433609_s_at detects both splice forms, whereas probe set 1453117_at detects only the minor Surf2 transcript. Note that the first two probes in probe set 1453117_at span the splice junction between exon 5 and exon 6.

The assignment of probe set 1427285_s_at to Surf4 is clearly an error. The Affymetrix NetAffx record (April 2004) for this probe set is inconsistent, with one section (Genomic Alignment of Target Sequence) showing that the target sequence for the probe set aligns to chromosome 19, but another section (Public Domain and Genome References, Chromosomal Location) showing that the Surf4 gene is on chromosome 2. The error probably comes from the GenBank record for the EST AI788623 [18], which Affymetrix gives as the representative sequence for this probe set (Target Description section) [7]. The GenBank record includes the terms 'similar to Mouse surfeit locus surfeit 4 protein gene' in the defline. An alignment of the AI788623 sequence to the UCSC March 2005 assembly of the mouse genome using BLAT [19] showed that the first 238 bases from the 5' end of the 569-base sequence align perfectly to chromosome 2, while the 335 bases starting from the 3' end align perfectly to chromosome 19. The probes for probe set 1427285_s_at align perfectly to chromosome 19, and to the part of the AI788623 sequence that maps to chromosome 19. The Bioconductor annotation method starts from AI788623. This is an EST and is not found in LocusLink [20]. Searching the UniGene mouse Build #139 Mm.data file [21] shows that AI788623 is in cluster Mm.300594, Surf4, which maps to LocusID 20932, Surf4.

### Observations of the expression of the eight probe sets matches the expectations from the annotation analysis

The Gene Expression Omnibus (GEO) [22] record GSE3749 is an 11-point time course study of differentiating J1 Embryoid Bodies in *Mus musculus *[23]. The experiment consists of triplicate observations, made at 0 hr, 6 hr, 12 hr, 18 hr, 24 hr, 36 hr, 48 hr, 4 days, 7 days, 9 days and 14 days. We calculated the Pearson correlation coefficient between different pairs of probe sets using the mean value at each time point.

**Figure 4:**
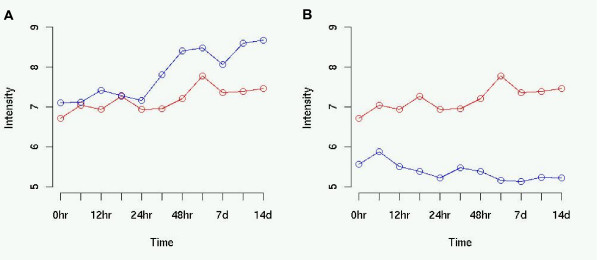
a) The positive correlation (0.77) between 1455822_x_at (red) and 1416213_x_at (blue). b) The negative correlation (-0.62) between 1433609_s_at (blue) and 1455822_x_at (red).

**Figure 5:**
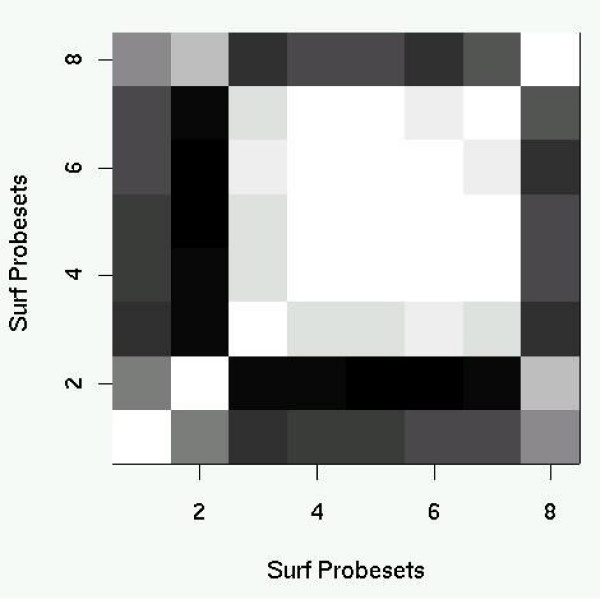
A greyscale matrix of correlation values for all the pairs of probe sets assigned to Surf4. The probe set order is 1453117_at and 1433609_s_at (Surf2), 1455822_x_at, 1416213_x_at, 1448255_a_at, 1434589_x_at, and 1436797_a_at (Surf4) and 1427285_s_at (Chromosome 19). Positive correlations are light grey to white and negative correlations are dark grey to black.

Figure 4a shows the positive correlation (+0.77) between the probe sets 1455822_x_at and 1416213_x_at, both of which map to Surf4. Such a high correlation is to be expected for two probe sets which map to the same transcript. Figure 4b shows the negative correlation between the probe sets 1455822_x_at and 1433609_s_at. We do not have sufficient understanding of the biology to know whether such a negative correlation is significant, but we would not expect such a negative correlation if the two probe sets are mapping to the same transcript. Figure 5 shows a grey-scale matrix of all the correlation values for all pairs within the 8 probe sets. The matrix shows that the two probe sets which map to Surf2 are correlated with each other, the five probe sets which map to Surf4 are all correlated with each other, but that the transcripts for Surf2 and Surf4 are not correlated. Furthermore, the false transcript on chromosome 19, 1427285_s_at, is more closely related in expression to Surf2 than it is to Surf4.

### Differential expression of rat probe sets mapping to the same gene

The RAE230A rat expression data was generated by members of the London Pain Consortium (LPC) or extracted from GEO [22]. The first LPC experiment measured differential expression in spinal cord tissue from a rat model of spinal long-term potentiation (LTP) under two conditions, sham operation and operation followed by stimulation sufficient to induce LTP (Tony Dickenson, Steve Hunt and Lars Rygh, unpublished), hereafter referred to as the LTP experiment. The second LPC experiment studied dorsal root ganglia of rat under two conditions, sham operation and operation followed by exposure to Nerve Growth Factor (Steve McMahon, unpublished), hereafter referred to as the NGF experiment. Record GSE2401, which we extracted from GEO, measures gene expression in rat kidney for two conditions, normo- and hypotensive animals [24], and is hereafter referred to as the kidney experiment. The rat datasets were chosen because the LPC experiments measure gene expression in related experiments, whereas GSE2401 measures gene expression from an unrelated experiment, and so this enabled us to see if the results for these different experiments bear any relation to each other. The CEL files were processed using GCRMA [25], and the sliding z-scores method of Quackenbush [26] was used to measure differential expression.

#### Results for the LTP experiment

1757 LocusIDs mapped to more than 1 probe set in the Bioconductor Mar/04 rae230a annotations. Variances in the z-scores of probe sets mapping to the same LocusID ranged from 5.00 × 10^-7 ^to 18.4, with 30 LocusIDs having z-score variances greater than 5.0. Table 3 shows the groups of probe sets resulting in the 10 largest z-score variances.

**Table 3:** LocusIDs and probe sets corresponding to the ten largest z-score variances for the LTP data set.

**Z-score Variance**	**LocusID**	**Gene Symbol**	**Probe set ID**	**Z-score**	**Affymetrix Annotation Grade (Apr/06) [7]**	**Comments**
18.4	24241	Calca/CGRP	1369116_a_at	-6.714	A	alt. splicing
			1369117_at	0.164	A	
			1370775_a_at	-7.701	A	
15.8	59329	Snf1lk	1368596_at	0.947	A	alt. poly(A)
			1368597_at	-4.67	A	
12.9	25513	Pik3r1	1370114_a_at	2.598	A	error
			1371776_at	-2.479	E	
12.6	316085	RGD1307844_predicted	1373605_at	0.712	A	indeterminate/error
			1373920_at	-4.315	E	
12.1	170796	Grin3b	1387559_at	-3.852	A	error
			1388905_at	1.058	A	
11.0	56827	Cacna1i	1369211_at	-4.51	A	alt. splicing
			1370641_s_at	0.173	A	
9.8	29415	Edg5	1367920_at	-4.581	A	alt. poly(A)
			1386989_at	-0.157	A	
9.1	192181	Podxl	1369895_s_at	1.951	A	alt. poly(A)
			1387933_s_at	-2.313	A	
9.0	311846	Lrrc8_predicted	1374296_at	-0.822	E	error?
			1382920_at	-5.071	A	1374296_at beyond 3' end of RefSeq
8.6	114124	Akap1	1369069_at	1.653	A	alt. splicing
			1388070_a_at	-2.494	A	

##### Alternative splicing

**Figure 6:**
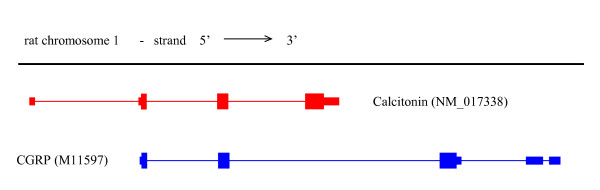
Alignment of the mRNAs for calcitonin (red) and CGRP (blue) to the genomic sequence (black line). The GenBank [28] accession numbers are given in parentheses. The coloured bars represent exons, with the narrower sections representing untranslated regions. The thin lines connecting the exons represent spliced out intronic regions. Genomic sequence was from the UCSC June 2003 rat assembly [10], and the alignments of the mRNAs to the genomic sequence were obtained from UCSC Genome Bioinformatics [10].

**Figure 7:**
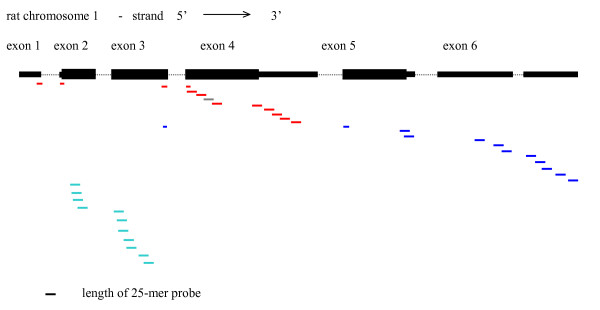
Mapping of individual probes in probe sets 1369117_at (red, grey lines), 1370775_a_at (blue lines) and 1369116_a_at (turquoise lines) to genomic sequence of the rat calcitonin alpha/CGRP gene. The arrow shows the direction of transcription. The introns are represented by black dotted lines and are not drawn to scale. The fifth probe in the 1369117_at probe set (grey line) does not match the genomic sequence perfectly – there is a single base mismatch between the probe sequence and the genomic sequence. Genomic sequence was from the UCSC June 2003 rat assembly [10].

LocusID 24241 had the largest z-score variance. LocusID 24241 maps to probe sets 1369116_a_at, 1369117_at and 1370775_a_at (Table 4), and corresponds to the rat calcitonin alpha/calcitonin gene-related peptide (CGRP) gene. The mammalian Calca/CGRP gene has six exons, and is considered a model gene for the study of alternative splicing [27]. Splicing together the first four exons produces the mRNA for calcitonin alpha, whereas splicing together exons one to three, five and six produces the mRNA for CGRP (Figure 6). The mRNA is processed in a tissue-specific manner to produce mainly the serum calcium-regulating hormone calcitonin in thyroid C cells, whereas in neuronal cells the major product is CGRP, a neuropeptide. As shown in Figure 7 the probes in probe set 1369116_a_at map to exons 2 and 3, which would be present in both calcitonin and CGRP transcripts. The probes in probe set 1369117_at map to exon 4, which would only be present in calcitonin mRNA, whereas the probes in probe set 1370775_a_at map to exons 5 and 6, which would only be present in CGRP mRNA. Note that the first probe in probe set 1370775_at maps to the splice junction between the 3' end of exon 3 and the 5' end of exon 5.

**Table 4:** Z-scores and gene localization of probe sets mapping to the 3 top ranked LocusIDs for the LTP data set.

**LocusID**	**Probe Set ID**	**Z-score**	**Region of gene covered by probes**
24241	1369116_a_at	-6.714	exon 2, exon 3
	1369117_at	0.164	exon 4
	1370775_a_at	-7.701	exon5, exon 6
59329	1368596_at	0.947	exon 15, exon 16
	1368597_at	-4.67	exon 14
25513	1370114_a_at	2.598	chr2, reverse strand
	1371776_at	-2.479	chr2, forward strand

Alignment of the target sequences to the UCSC June 2003 rat genome assembly was performed using BLAT [19].

##### Alternative polyadenylation sites

**Figure 8:**
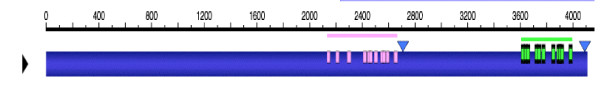
Screen shot of the Affymetrix probe set display tool showing the consensus sequence for probe sets 1368596_at and 1368597_at (blue bar) [29]. The arrow at the left shows the 5' to 3' direction along the consensus sequence. The pink bars represent the 1368597_at probes, and the green bars the 1368596_at probes. The blue triangles above the consensus sequence show poly(A) sites.

The LocusID with the second highest z-score variance was LocusID 59329, with a z-score variance of 15.8. LocusID 59329 corresponds to the rat SNF1-like kinase (Snf1lk) gene and maps to probe sets 1368596_at and 1368597_at (Table 4). The consensus sequences for the two probe sets are identical (Figure 8). The probe sets are about 1000 bp apart, and were designed to detect transcripts produced using alternative polyadenylation sites.

##### Adjacent genes on opposite strands

**Figure 9:**
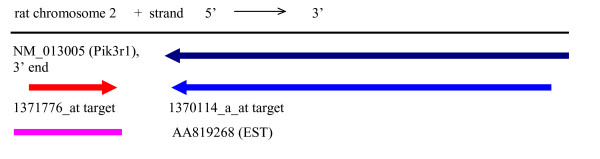
Alignment of the target sequences for probe sets 1370114_a_at and 1371776_at to the genomic sequence of rat chromosome 2. The arrowheads show the direction of transcription. Introns are not shown, and only the 3' end of the Pik3r1 sequence is shown. Target sequences were obtained from Affymetrix [7]. Transcript sequences were obtained from GenBank [28]. Genomic sequence of rat chromosome 2 and genomic alignments of the transcripts to the UCSC June 2003 rat assembly were obtained from UCSC Genome Bioinformatics [10].

The LocusID with the third highest z-score variance was LocusID 25513, with a z-score variance of 12.9. LocusID 25513 maps to probe sets 1370114_a_at and 1371776_at (Table 4) and corresponds to the gene phosphatidylinositol 3-kinase, regulatory subunit, polypeptide 1 (Pik3r1). However, the target and consensus sequences for probe set 1370114_a_at align to the reverse strand of chromosome 2, whereas the target and consensus sequences for probe set 1371776_at align to the forward strand of chromosome 2 (Figure 9), so the two probe sets cannot possibly represent the same gene. Thus the difference in z-scores is due to the fact that the two probe sets are measuring different genes, rather than different transcripts from the same gene.

The Affymetrix NetAffx record (June 2005) [7] for probe set 1371776_at warns that this is an E-grade annotation, based only on ESTs. The assignment was obtained from the current UniGene cluster containing the GenBank EST AA819268, the Representative Public ID for this probe set.

#### The results for the NGF and Kidney experiments

Table 5 shows the groups of probe sets resulting in the 10 largest variances for the NGF experiment. Table 6 shows the groups of probe sets resulting in the 10 largest variances for the kidney experiment. Each of these annotations was assessed by hand, as described for the LTP experiment, and the detailed breakdown of the causes of the discrepancies are listed in Tables 5 and 6.

**Table 5:** LocusIDs and probe sets corresponding to the ten largest z-score variances for the NGF data set.

**Z-score Variance**	**LocusID**	**Gene Symbol**	**Probe set ID**	**Z-score**	**Affymetrix Annotation Grade (Apr/06) [7]**	**Comments**
103.8	25513	Pik3r1	1370114_a_at	-8.577	A	error
			1371776_a_at	5.83	E	
33.4	363087	RGD1560364_predicted	1376129_at	3.837	E	error, opposite strands
			1380038_at	-4.34	B	
17.0	298530	RGD1308209_predicted	1370876_at	1.788	C	error, different chromosomes
			1376841_at	-4.046	E	
13.6	24737	RT1-Aw2 (MHC Class I antigen)	1369110_x_at	1.447	A	
			1370428_x_at	2.057	A	
			1370429_at	3.397	A	
			1370463_x_at	-4.724	-	
			1370972_x_at	0.573	-	
			1371078_at	3.449	B	
			1371111_at	0.515	-	
			1371119_at	0.796	-	
			1371171_at	-0.398	A	
			1371209_at	7.035	-	
			1371210_s_at	1.582	A	
			1371213_at	1.965	-	
			1388071_x_at	4.308	A	
			1388202_at	-7.565	A	
			1388203_x_at	-6.808	B	
			1388236_x_at	-0.588	-	
			1388254_a_at	-0.57	-	
			1388255_x_at	4.886	-	
			1388256_at	-0.335	-	
			1388694_at	5.334	-	
			1389734_x_at	0.967	B	
13.2	116561	Cltb	1367907_a_at	1.71	A	error, 1375324_at, wrong strand
			1375324_at	-3.437	E	
13.0	171454	Btbd14b	1371826_at	2.191	E	1371826_at, 1.5 kb beyond 3' end of RefSeq sequence
			1387443_at	7.287	A	
12.9	24225	Bdnf	1368677_at	6.115	A	alt. poly(A) sites;
			1368678_at	1.034	A	1368678_at last 4 probes don't align to genomic sequence
12.2	311846	Lrrc8_predicted	1374296_at	1.116	E	error
			1382920_at	6.06	A	
12.1	64160	Basp1	1369310_at	3.803	A	1375143_at – error, wrong strand;
			1375143_at	9.602	E	
			1398350_at	3.391	C	1398350_at – beyond 3' end of RefSeq
11.8	25601	Oprm1	1369109_at	4.617	A	alt. splicing
			1387461_at	-0.235	A	

**Table 6:** LocusIDs and probe sets corresponding to the ten largest z-score variances for the kidney data set.

**Z-score Variance**	**LocusID**	**Gene Symbol**	**Probe set ID**	**Z-score**	**Affymetrix Annotation Grade (Apr/06) [7]**	**Comments**
39.7	290851	Vps36_predicted	1373423_at	8.39	E	1373423_at, consensus sequence 2.0 kb downstream from 3' end of Ensembl prediction for Vps36
			1389191_at	-0.52	B	
37.5	79131	Fabp3	1367660_at	-0.84	A	1376522_at maps to intron
			1376522_at	7.82	E	
32.4	29223	Ak3l1	1371824_at	-0.51	B	1371824_at, 9 probes are beyond 3' end of RefSeq sequence
			1398285_at	-8.55	A	
29.0	293949	RGD1310475_predicted	1375560_at	-3.29	A	error, opposite strands; 1389839_at, wrong strand;
			1389839_at	4.33	E	
23.4	171522	Cyp2d22	1370329_at	8.72	A	alt. poly(A);
			1387913_at	1.87	A (9/11)	1387913_at only 9 probes align to the RefSeq transcript
18.4	289696	LOC289696	1371189_x_at	0.50	B	error;
			1371190_at	7.97	B	1371190_at is on opposite strand from the other 2; consensus sequences align to 5 places in genome
			1388244_s_at	0.58	A	
16.9	25715	Slc11a2	1367877_at	-0.49	B	alt. splicing
			1388059_a_at	-6.31	A	
15.5	171099	Rasd2	1370372_at	4.27	A	alt. poly(A)
			1370373_at	-1.29	A	
14.7	287005	Camk2n1	1370853_at	4.31	A (9/11)	error;
			1374307_at	-0.37	E	1389876_at, wrong strand; 1374307_at, beyond 3' end of RefSeq; 1370853_at, only 9 probes align to the RefSeq transcript
			1389876_at	-3.28	E	
14.6	25197	St6gal1	1370714_a_at	-6.54	A	alt. poly(A)
			1370907_at	-1.13	A	

#### Rank comparisons between the RAE230A experiments

Tables 3, 5 and 6 illustrate the ten largest discrepancies in differential expression for probe sets mapping to the same LocusID for three different experiments. In each case we are able to assess a likely cause for the discrepancy, whether it is based on biological phenomena such as alternative splicing and polyadenylation or annotation errors.

Phenomena such as alternative splicing or polyadenylation need to be tightly regulated on a genomic scale. We therefore expect that it be may possible to discover evidence for co-regulated splicing and polyadenylation for different sets of genes. We have made a first search for co-regulated families of probe sets by cross-comparing the ranks in three different experiments. Table 7 shows one such comparison, the top ten ranked LocusIDs in the LTP data set, compared with their ranks in the NGF and kidney data sets. The two top ranked LocusIDs in the NGF data set were 25513 (Pik3r1) and 363087 (RGD1560364_predicted), respectively, while the two top ranked LocusIDs in the kidney data set were 290851 (Vps36_predicted) and 79131 (Fabp3), respectively. Two LocusIDs, 25513 and 311846, whose variances in differential expression are due to annotation errors, were found to be common to the LTP and NGF experiments, and this provides some support for our search strategy. However, we have found no evidence in support of co-regulated splicing and polyadenylation between different genes, but we acknowledge that our dataset (only three experiments) may be too limited to reliably perform this task.

**Table 7:** The ranks of the LocusIDs with the ten highest z-score variances for the LTP data set compared against their ranks in the NGF experiment and the kidney experiment.

**LocusID**	**Rank in LTP**	**Rank in NGF**	**Rank in kidney**
24241	1	22	1509
59329	2	1357	1544
25513	3	1	89
316085	4	1009	187
170796	5	137	878
56827	6	1675	1448
29415	7	727	928
192181	8	418	741
311846	9	8	40
114124	10	181	1573

## Discussion and Conclusion

The goal of biologists performing gene expression microarray experiments is to obtain a list of genes that are upregulated or downregulated under particular conditions. Analysis of Affymetrix microarray data yields expression values for probe sets, which are converted into expression values for genes by the probe set annotations. Certain genes are represented by two or more probe sets on Affymetrix GeneChips, and it could be naively assumed that the multiple representations should all tell a consistent picture, i.e. they will all indicate that the gene is either up-regulated, down-regulated or unchanged. However, our analysis shows that the reality is actually more complicated, and perhaps more interesting than the naïve assumption.

Multiple probe sets assigned to the same gene were found to detect cases of alternative splicing, use of alternative poly(A) sites, or errors. These conclusions were reached from the examination of the eight probe sets mapped to the mouse Surf4 gene. We have also compared the differential expression in three different rat experiments, and in each case we have found a number of probe sets which are assigned to the same gene but show a variety of differential expression changes some of which are inconsistent (up versus down). Furthermore, in some cases one probe set measures more than one transcript. For example, in the case of the three probe sets mapping to the rat calcitonin/CGRP gene, probe set 1369116_a_at detects both splice forms. For this particular probe set, it is therefore possible that it will detect multiple transcripts, whose relative abundance will differ under distinctive biological conditions. It is therefore likely that other experiments will also find similar effects, and for different genes to the ones we describe here. We recommend that as a matter of course, it should not be assumed by an experimentalist that multiple representations of the same gene are actually measuring the same transcript. Each probe set should be treated independently and should not be averaged, or used in any way which assumes that they are different samples from an underlying uniform population. Our findings are in close agreement with other similar analyses of probe set mappings [30-33]. The analysis of Harbig et al. [31] suggests that over one-third of probe sets on the HG-U133 plus 2.0 GeneChip array detect multiple transcripts, suggesting that accurate annotations are needed before GeneChip data can be reliably interpreted. The novelty of our work is in the use of differential expression measures as a source of data with which to benchmark annotations.

Frequently the lists of genes observed to be differentially expressed are eye-balled by the experienced biologist familiar with the literature on earlier experiments. Indeed, a gene for assessing the quality control of the LTP experiment is Calcitonin/CGRP, which shows evidence for alternative splicing, and in the NGF experiment Brain-derived neurotrophic factor (Bdnf), which shows evidence for alternative polyadenylation and Opioid receptor mu 1 (Oprm1), which may be undergoing alternative splicing. The analysis we describe here may help to circumvent the possibility that an experienced experimentalist will discard the results because the list of differentially expressed genes contains a name that should not be there (false positive) or doesn't contain a name that should be there (false negative). Our analysis indicates that GeneChips are measuring a range of biological phenomena, and certainly much more than their standard use as a surrogate for determining which proteins are most likely to be differentially regulated. Indeed, it is important to better include our knowledge of the transcriptome, before we interpret GeneChip data in terms of a protein measure. In particular, alternative splicing may result in different protein isoforms, and so it should be established which particular isoform is of interest to the experimentalist, before determining which probe set, or subset of probes, provide the best diagnostic of the mRNA that will go on to generate the protein.

Alternative polyadenylation does not modify the form of the protein, but it does act to change the structure of the 3' UTR, as indeed does alternative splicing. The 3' UTR frequently contains motifs related to mRNA control and regulation [34], and so transcripts undergoing differential polyadenylation, and splicing, may actually modify the expression rates and location of the corresponding protein. GeneChips contain probes falling on separate sides of a polyadenylation signal, and so, in principle, GeneChips can be used as a discovery tool for finding families of co-regulated polyadenylation decisions. However, to our knowledge, nobody has reported such a use in the literature.

Our knowledge of the transcriptome is rapidly evolving and it appears that genomes in higher eukaryotes, at least, produce a range of overlapping transcripts and chimeras [35,36]. The design of Affymetrix GeneChips occurred prior to this knowledge, but it made heavy use of EST databases which will have included transcripts with the exotic forms that are now being re-discovered. This may account for why probes are frequently found to align beyond the RefSeq sequence for the corresponding gene. It is therefore likely that Affymetrix GeneChips have the potential to be used as a discovery tool for exploring the exotic transcriptome, and go beyond their standard use as a measure of mRNA for protein-coding genes. To our knowledge, there has been little use of GeneChips in this way, but given that there are now large repositories of GeneChips in GEO [22] and other repositories, a systematic survey of probes not corresponding to RefSeqs may discover interesting biological phenomena associated with the exotic transcriptome.

## Methods

### GeneChip Annotations

#### Affymetrix

The annotation method used by Affymetrix prior to June 2004 [6] was based on UniGene [4]. During the array design process mRNA and EST sequences obtained from UniGene were re-clustered and one high quality sequence was chosen to represent each cluster. The genomic location, gene symbol and LocusID for a probe set were obtained from the UniGene cluster containing the representative sequence for that probe set. Additional information, including RefSeq accession numbers, was obtained from LocusLink [37].

The new Affymetrix annotation method introduced in October 2004 [38] uses mRNA sequences obtained from public databases and clusters them to 90% sequence identity using BLAT [19]. The longest sequence in each cluster is used as the representative for that cluster, with preference given to RefSeq sequences. Pair-wise alignment of the probe sequences against the non-redundant mRNA set is used to assign probe sets to transcripts. Annotations are described as Class A, B or C depending on the number of probes which align exactly to the transcript [38]. A fourth type of transcript assignment based only on EST evidence, called an E-grade annotation, was introduced by Affymetrix in March 2005.

#### Bioconductor

The Bioconductor [8] annotation packages are built using the Bioconductor package AnnBuilder [39,40]. The starting points for the annotations are the mappings of probe set identifiers to GenBank accession numbers provided by Affymetrix. The first step is to map the GenBank accession numbers to LocusIDs by combining data available from LocusLink, UniGene and other sources of information. The unified mapping that results is assumed to be more reliable. When the various sources provide conflicting mappings, the mapping from LocusLink is used. The LocusLink record provides gene name, gene symbol, chromosomal location, UniGene ID and RefSeq accession number.

### Data

#### The Affymetrix MOE430A and RAE230A arrays

The MOE430A array [2] was designed using the Mouse Genome Sequencing Consortium [41] version 3 assembly, April 2002. Clustering information used in the design of the array was from UniGene Build 107. The RAE230A array [3] was designed using a preliminary draft assembly of the rat genome (June 2002) [42]. Clustering information used in the design of the array was from UniGene Build 99.

#### Genomic sequence and chromosomal coordinates

Mouse genomic sequence and chromosomal coordinates were obtained from UCSC Genome Bioinformatics [10,13,14]. The UCSC October 2003 mm4 *Mus musculus *genome assembly is based on the NCBI Build 32 assembly, the UCSC May 2004 mm5 assembly is based on NCBI Build 33 and the UCSC March 2005 mm6 assembly is based on NCBI Build 34. Rat genomic sequence and chromosomal coordinates were obtained from UCSC Genome Bioinformatics. The UCSC rn3 June 2003 *Rattus norvegicus *genome assembly is based on version 3.1 produced by the Atlas group at Baylor Human Genome Sequencing Center (HGSC) as part of the Rat Genome Sequencing Consortium [42].

Chromosomal coordinates of individual probes from the MOE430A and RAE230A arrays were obtained from Ensembl version 28 (Feb. 2005) [15], using AffyProbe [43] and the respective Affymetrix probe set identifier. The Ensembl version 28 *Mus musculus *assembly was based on NCBI Build 33 of the mouse genome.

#### NCBI data files

The LocusLink LL_tmpl January 27, 2004 file was obtained from NCBI [20]. The UniGene *Mus musculus *Build #139 (July 13, 2004) and Build #140 (August 18, 2004) Mm.data files were obtained from the NCBI ftp repository [21].

#### Affymetrix files

Affymetrix annotation files for the MOE430A and RAE230A arrays were from the June 2004 through April 2006 Affymetrix quarterly releases. Annotation files and target and consensus sequence files were obtained from Affymetrix [11,44]. Sequences and annotations for individual probe sets were also obtained from the NetAffx Analysis Center [6,7] and were from the April 2004 quarterly release as well as the releases listed above.

#### Bioconductor files

The Bioconductor [8] moe430a and rae230a packages [9] were run under R, an open source language and environment for statistical computing [45]. The version 1.5.1 (March 2004) moe430a and rae230a packages were run under R version 1.9.1 on the Mac OS X operating system. The Bioconductor version 1.6.5 (September 2004) and version 1.7.0 (January 2005) moe430a and rae230a packages were run under R-2.0, moe430a version 1.8.5 (May 2005), rae230a version 1.8.4 (May 2005), and moe430a and rae230a version 1.10.0 (October 2005) were run under R-2.1, and moe430a and rae230a version 1.12.0 (April 2006) were run under R-2.3.

#### MOE430A expression data

The MOE430A data was extracted from the GEO database [22]. We studied record GSE3749, which is an 11-point time course study of differentiating J1 Embryoid Bodies in *Mus musculus*. The experiment consists of triplicate observations, made at 0 hr, 6 hr, 12 hr, 18 hr, 24 hr, 36 hr, 48 hr, 4 days, 7 days, 9 days and 14 days. The data were submitted by the Ontario Genomics Innovation Centre (OGIC) and is part of the StemBase repository [23]. The CEL files were processed using GCRMA [25], and we calculated the Pearson correlation coefficient on the mean of the triplicates.

#### RAE230A expression data

The RAE230A data was generated by members of the LPC or extracted from GEO [22]. The LTP experiment measured spinal cord tissue from a rat model of spinal long-term potentiation under two conditions, sham operation and operation followed by stimulation sufficient to induce LTP (Tony Dickenson, Steve Hunt and Lars Rygh, unpublished). The NGF experiment studied dorsal root ganglia of rat under two conditions, sham operation and operation followed by exposure to Nerve Growth Factor (Steve McMahon, unpublished). The kidney experiment was record GSE2401 from GEO, which measures gene expression in rat kidney for two conditions, normo- and hypotensive animals [24]. The CEL files were processed using GCRMA [25], and the sliding z-scores method of Quackenbush [26] was used to measure differential expression.

## Abbreviations

alt. alternative

defline definition line

CGRP calcitonin gene-related peptide

EST Expressed sequence tag

ID Identifier

GEO Gene Expression Omnibus

GeneID Entrez Gene Identifier

LocusID LocusLink Identifier

LPC London Pain Consortium

LTP Long-term potentiation

NCBI National Center for Biotechnology Information

OGIC Ontario Genomics Innovation Centre

RefSeq Reference Sequence

UCSC University of California at Santa Cruz

UTR Untranslated region

## Authors' contributions

AH proposed this study and provided the analysis of the microarray data. MS performed the bioinformatics analysis of the genes of interest. Both authors read and approved the final manuscript.
